# Micronutrient Status in 153 Patients with Anorexia Nervosa

**DOI:** 10.3390/nu9030225

**Published:** 2017-03-02

**Authors:** Najate Achamrah, Moïse Coëffier, Agnès Rimbert, Jocelyne Charles, Vanessa Folope, André Petit, Pierre Déchelotte, Sébastien Grigioni

**Affiliations:** 1 Nutrition Department, Rouen University Hospital, 76183 Rouen, France; moise.coffier@chu-rouen.fr (C.M.); agnes.ribert@chu-rouen.fr (R.A.); jocelyne.charle@chu-rouen.fr (C.J.); vanessa.folope@chu-rouen.fr (F.V.); andre.petit@chu-rouen.fr (P.A.); pierre.dechlotte@chu-rouen.fr (D.P.); sebastien.grigioni@chu-rouen.fr (G.S.); 2 INSERM Unit 1073, 76183 Rouen, France; 3 Institute for Research and Innovation in Biomedicine, Normandie Université, 76183 Rouen, France; 4 Clinical Investigation Centre CIC 1404 INSERM, 76183 Rouen, France

**Keywords:** micronutrients, anorexia nervosa

## Abstract

Micronutrient status in Anorexia Nervosa (AN) has been poorly documented and previous data are often contradictory. We aimed to assess micronutrient status in a large population of AN patients. The relationships between micronutrient status and body composition were also determined. Anthropometric, biochemical parameters and body composition data were collected at referral in 153 patients with AN (28.5 ± 11 years). At least one trace element deficit was observed in almost half of patients; the most frequent was selenium deficit (40% of patients). At least one vitamin deficit was observed in 45.7% of patients, mostly vitamin A and B9. Albumin, transthyretin and CRP were within normal range in most patients. No correlations were found between body composition and micronutrient status. Our study suggests that micronutrient status is often altered in AN patients, which may contribute to neuropsychiatric dysfunction. Monitoring of micronutrients and correction of deficits should be included in the routine care of AN patients.

## 1. Introduction

Anorexia nervosa (AN) is an eating disorder characterized by a significant malnutrition (more than 15% BMI deficit), a fear of gaining weight, and an excessive obsession about body shape and weight. A disturbed body image perception is often associated, as well as denial of troubles [1]. Two subtypes have been described, the pure restrictive subtype (AN-R), and the binge–purging subtype (AN-BP) with recurrent binge eating and purging through self-induced vomiting, laxative misuse or other purge maneuvers. The prevalence rate of AN has steadily increased over the past decades, and is as high as 2%–3% in adolescents and young adults [2]. A mortality rate of 5%–10% at 10 years has been reported, making AN the psychiatric disorder with the highest mortality [3], although more recent studies report less severe mortality rates [4]. AN is associated with significant psychiatric comorbid conditions, including anxiety, depression, obsessive–compulsive disorders and excess physical exercise referred as hyperactivity [5,6]. Somatic complications related to malnutrition including bradycardia, hypokaliemia, hypotension, anemia, hormonal imbalance, and osteoporosis [7] are well documented in AN. All these psychiatric and somatic complications massively impair functional capacities and quality of life [8]. The pathophysiological mechanisms of AN remain debated, but evidence is accumulating for a dysregulation of neuropeptidergic regulation of eating behavior in response to different types of stress [9,10].

Whatever the mechanisms implicated in the initiation of AN, this disorder is characterized by a major imbalance between reduced dietary intake of various macro- and micronutrients and expenses that may be increased by pathological features such as sleep deprivation and hyperactivity. Only few studies until now have evaluated micronutrient (vitamins and element trace) status in patients with AN, with conflicting results [7,11,12,13,14,15,16]; some of these studies evaluated micronutrient dietary intake based on questionnaires (with wide uncertainty on the reliability of declarations), while others reported plasma levels. In one study, no micronutrient deficiency was reported [17]. However, clinical and biological evidence of skin lesions, severe bone demineralization, deficit neuropathy or anemia are suggestive of micronutrient deficits. Accordingly, iron and zinc deficiency have been frequently described in adolescents [11,12,13]. A thiamin deficit was found in 19% of a small group of adult AN patients [14]. Conflicting data on vitamin A and beta-carotene levels have been reported [15,16]. Declared dietary intakes of vitamins A, K, most B vitamins, calcium and vitamin D were found to be higher in patients with AN and closer to Dietary Reference Intake than in healthy adolescents [18]. The validity of such studies is probably jeopardized by a high and ill-defined proportion of self-supplementations of patients with different nutritional supplements.

Having a reliable evaluation of micronutrients status in AN patients is strongly needed to improve nutritional care and optimize the metabolic responses during refeeding, especially at the early stages of rehydration and carbohydrate supply where electrolytes and micronutrients needs are massively increased, which a high risk of inappropriate refeeding syndrome if adapted monitoring and supplementation is not implemented [19]. In later stages of refeeding, suboptimal correction of micronutrients needs may also limit nutritional and neurocognitive restoration, due to sustained oxidative stress among other mechanism [20].

The aim of this retrospective study was thus to assess the micronutrient initial status of a large population of AN patients referred in a regional reference Eating Disorder Centre.

## 2. Material and Methods

### 2.1. Study Design and Patients Selection

This retrospective study included all consecutive women patients with AN referred to the Department of Clinical Nutrition (University Medical Center, Rouen, France) during the 2009–2011 period. Both restricting subtype (AN-R) and binge–purging subtype (AN-BP) were included. All women patients with AN aged >17 years meeting at that time the diagnostic criteria from the Diagnostic and Statistical Manual of Mental Disorders, fourth edition (DSM-IV) were included, and were evaluated according to routine procedures on a day-hospital basis. Every patient has been evaluated for weight, height, Body Mass Index (BMI), micronutrient status and body composition (FM (Fat Mass) and FFM (Fat Free Mass)). To establish a baseline micronutrient intake, all the included patients were not previously treated and none declared to take prescribed or self-administered micronutrients supplements. All patients agreed to benefit from the routine clinical and biological evaluation set up as standard in the Department for all malnourished patients.

### 2.2. Clinical Data

Weight and height were measured under standardized conditions, by the same operator, in the morning, after a fasting period of 12 h, in light clothes without shoes. BMI was calculated as body weight divided by squared height (kg/m^2^). Usual clinical features of malnutrition were recorded in the charts (such as bradycardia, hypotension, edema, etc.).

### 2.3. Biological Data

After a 12-h fasting period, biochemical analyses were performed from venous blood samples in the hospital’s central laboratory with routine methods, using reference measurements from the Department of Clinical Biochemistry. Plasma concentrations of the following proteins were determined: albumin (N: 35–52 g/L), transthyretin (N: 0.20–0.45 g/L), CRP (N: <5 mg/L). Trace elements and electrolytes profile included Zinc (N: 9–17 μmol/L), Copper (N: 10–40 mcmol/L), Selenium (N: 0.90–1.65 mcmol/L), Magnesium (N: 0.75–1.00 mmol/L), Phosphorus (N: 0.87–1.50 mmol/L), and Calcium (N: 2.15–2.55 mmol/L). Plasma levels of vitamin A (N: 430–800 mcg/L), E (N: 7–17 mg/L), B9 (N: 10.4–42.4 nmol/L), and B12 (N: 141–489 pmol/L) were also analyzed.

### 2.4. Body Composition

Body composition was determined using multifrequency bioelectrical impedance analysis (BIA, Bodystat Quadscan 4000) as previously described [21,22] and according to the manufacturer’s recommendations, allowing assessment of fat free mass (FFM) and fat mass (FM).

### 2.5. Statistical Analysis

All statistical analyses were performed using SPSS version 10.0 (SPSS Inc., Chicago, IL, USA). Continuous data were presented as the mean ± standard deviation (SD) and means comparison were performed using Student’s *t*-test. Categorical data were presented as count and percentage (%) and percentage comparison were performed using Chi2 test followed by Fisher’s test for *n* < 5. Pearson analysis was used to analyze correlations between the groups. *p* < 0.05 was considered statistically significant difference.

## 3. Results

### 3.1. Anthropometric Data

A total of 153 patients were involved in this study, only women, mean age 28.5 ± 11 years. The mean disease duration before admission was 7.5 ± 8.4 years. Of the 153 patients, 91 (59.5%) presented with the restrictive (AN-R) subtype, and 62 (40.5%) with the binge–purging (AN-BP) subtype. Mean weight and BMI were 42.2 ± 5.3 kg and 17.4 ± 2.6 kg/m^2^, respectively. Patients with AN-R were more severely malnourished than AN-BP (Table 1). All this patients were followed as outpatients, and only a minority needed hospitalization after the initial screening.

### 3.2. Biological Parameters

Albumin (46 ± 4.6 g/L), transthyretin (0.26 ± 0.54 g/L) and CRP (2.1 ± 9 mg/L) levels were in normal range for most patients, with no difference between AN-R and AN-BP (online supplement table). Albumin level was notably reduced and CRP elevated in a few patients with the lowest BMIs.

**Figure 1:**
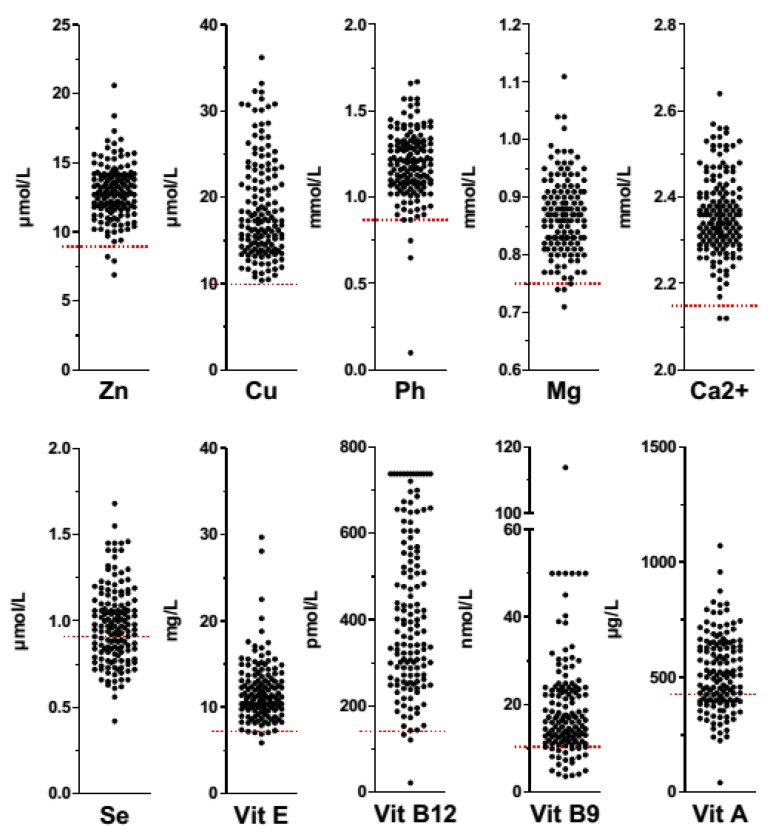
Micronutrients status in AN patients. Zinc (Zn), copper (Cu), phosphorus (Ph), magnesium (Mg), calcium (Ca2+), selenium (Se), vitamin E (Vit E), vitamin B12 (Vit B12), vitamin B9 (Vit B9), and vitamin A (Vit A) values represented by plots are expressed in µmol/L (Zn, Cu, and Se), mmol/L (Ph, Mg, and Ca2+), mg/L (Vit E), pmol/L (Vit B12), nmol/L (Vit B9) or µg/L (Vit A). Low micronutrients concentrations are represented by red lines. Plots under the red lines represent micronutrient deficiencies in AN patients.

Figure 1 shows individual data for micronutrients concentrations in AN patients. Mean plasma concentrations of zinc, copper, phosphorus, magnesium and calcium of the total population were within normal range (Table 2). However, a selenium deficit was present in 58 (40.6%) patients, and at least one trace element deficit was present in 43.7% of patients (Table 3).

The most prevalent deficits were for vitamin B9 and vitamin A. Other vitamin concentrations were in normal range in almost all patients. However, at least one vitamin deficiency was observed in 45.7% of patients (Table 3).

When micronutrients status was compared between subgroups of AN, no significant differences in micronutrient status were observed, excepted for a lower Vitamin E plasma concentration in the AN-R subgroup (*p* < 0.05) (Table 2). Moreover, no significant differences were observed in micronutrients deficiency between subgroups of AN.

### 3.3. Body Composition

According to multifrequency bioelectrical impedance analysis, Fat Free Mass (FFM) was 79.2% ± 10.5%m whereas Fat Mass (FM) was 19.5% ± 6.3% with significantly higher FFM in AN-R patients (*p* < 0.05 vs. AN-BP, Table 1). Inversely, Fat Mass (FM) was higher in AN-BP patients (*p* = 0.054 vs. AN-R, Table 1). No correlations were found between body composition and micronutrient status.

## 4. Discussion

The present study is so far the largest available on micronutrients status in AN patients evaluated before initiation of treatment and taking into account the subtype of AN. Roughly one half of patients present with at least one deficit in trace element and/or vitamin. Surprisingly at first glance, the other half of patients present with normal micronutrients concentrations despite severe malnutrition witnessed by a mean 20% of weight loss. Plasma proteins used routinely for the assessment of nutritional and inflammatory status were also analyzed.

In our study, plasma concentrations of usual nutritional protein markers, albumin and transthyretin, were normal in most patients despite a low BMI. This confirms other reports that albumin and CRP plasma concentration remains usually normal during AN, even in severely malnourished patients [23,24,25,26]. Mechanisms of preservation of albumin plasma concentration are not fully understood. Hemoconcentration due to dehydration especially in AN-BP patients may falsely normalize albumin level, with unmasking of hypoalbuminemia after rehydration [27]. On the other hand, anemia and reduced plasma volume are common in severely malnourished patients with AN; thus, even if albumin hepatic synthesis was marginally reduced, this may match with the reduction of plasma volume and lead to apparently normal concentration. In the absence of systemic inflammation, hepatic albumin synthesis and albumin degradation may not be significantly modified in AN, but precise isotope studies of albumin synthesis rate are lacking. It is generally considered that chronic and progressive malnutrition during AN is associated with an adaptative decrease of whole body protein breakdown, which may also include a reduction of albumin degradation. However, the only available study on albumin degradation during AN showed that albumin fractional and absolute degradation rate remained unaffected [28]. In the latter study, performed during refeeding, a large expansion of albumin extravascular pool was observed, which is well in accordance with the frequent occurrence of transient edema at the initiation of refeeding. However, albumin plasma level remains generally stable, which means that expansion of the pool is replenished by enhanced hepatic synthesis. Thus, maintenance of albumin plasma level during AN is common, and should not be considered as falsely reassuring by patients and clinicians. Plasma albumin may decrease only at advanced stages of AN-related cachexia, usually during phases of rapid worsening of weight loss with added complications such as pulmonary infections, diarrhea or profound pressure ulcers. At that stage, inflammation reflected by increased CRP is also common, and this acute phase reaction in AN patients should be considered as a strong marker of severity and complications risk.

Intake of many micronutrients is reduced in patients with AN [29,30] and a number of micronutrient deficiencies have been identified. Selenium deficiency was the most frequent deficit observed in our study. Selenium plays an important role as cofactor of antioxidant systems such as the glutathione peroxidase and is also involved in protection against infection, myocardial function and regulation of anxiety and mood [31]. Selenium acts synergistically with other antioxidant micronutrients such as vitamin C, E and carotenoids. Chronic malnutrition is known to lead to an antioxidant deficit, and increased oxidative stress in patients with AN has already been documented [32], with an impaired detoxifying capacity of reactive oxygen species via glutathione [33]. In addition to selenium depletion, reduced concentrations of erythrocyte tocopherol (vitamin E) could contribute to oxidative damage in AN [34]. Increased oxidative stress in the central nervous system has been implicated in the constitution and perpetuation of anxiety and depression [35], which are frequent comorbid conditions in AN [6]. In a recent study, nutritional improvement in patients with AN was followed by a restoration of antioxidant capacity and catalase antioxidant system [36]. Moreover, it was confirmed in a recent meta-analysis which reported that oral refeeding, even without full-weight restoration, improves oxidative stress in AN patients [20]. Thus, initial screening for selenium deficiency and careful supplementation during refeeding is warranted in AN patients to reduce oxidative stress.

Zinc deficiency was not observed in our study. This is in accordance with a former study in small group of patients [37]. However, other authors suggested that zinc deficiency could be a causative or perpetuation factor in AN [38]. Indeed, zinc deficiency may cause taste alterations and contribute to a variety of neuropsychiatric symptoms [39,40]. Some early reports supported the benefit of zinc supplementation on clinical outcome of AN [41,42], which was confirmed in a randomized, double-blind, placebo-controlled trial: use of zinc supplements (15 mg bid) improved the rate of weight gain by an unknown mechanism [43]. Zinc has been reported to be an appetite stimulator and to play also a role to limit the progression of cachexia and sarcopenia in other disease conditions [44]. It has also been proposed that low zinc intake may adversely affect neurotransmitters such as gamma-amino butyric acid (GABA) in different parts of the human brain and affect amygdala functions [45], an important structure for the central regulation of the autonomic nervous system, which is often affected in AN [46,47]. Thus, despite an incomplete understanding of the mechanisms underlying the actions of zinc, oral supplementation (around 14 mg daily) during refeeding should be considered as an integrative part of treatment for AN, at least during the first two months [45].

In our study, we did not find any correlation between body composition and micronutrient status. Despite a major reduction of fat mass, and restricted fat intake in AN patients, deficit of the lipophilic vitamins A and E were not commonly observed in our patients group, probably due to the high previous storage in this western population. Limited data in the literature state that, despite severe malnutrition, bioavailability of oral ergocalciferol in young women with AN was similar to that in healthy controls [48]. Deficit in vitamin B9 was found in 30% of patients, in relation with rapid depletion of the limited hepatic folate pool, which may contribute to the high frequency of anemia, together with iron deficiency.

This present study is retrospective and cross-sectional. Thus, it is not possible to assess whether micronutrients status normalize after weight recovery. Few previous studies focused on the micronutrient status change after weight gain in AN. Rock et al. found a normalization of vitamin abnormalities in 13 patients after refeeding (in about 2–6 weeks) [49]. This study involved only AN-BP patients (*n* = 8). McClain et al. reported that zinc supplementation increased plasma zinc level significantly in 33 eating-disordered patients (including both bulimia and AN) hospitalized, in comparison to patients without supplementation [12]. More recently, Castro et al. studied biochemical parameters at admission and after refeeding in 61 AN patients (52 AN-R and 9 AN-BP) [50]. At admission, folate and zinc deficits were the most frequent. Surprisingly, after refeeding, folate decreased significantly while zinc increased but did not reach normal value. Further studies are needed to assess micronutrient change after weight gain in AN.

Limitations of our study include the lack of biological measurements of antioxidant capacities that may correlate with selenium or zinc deficiency. Iron status was assessed only in the presence of anemia and therefore data are available only in a subset of patients (not shown). The population of patients studied was of intermediate severity, most of patients being followed ambulatory; it is expected that micronutrients status would have been more altered in patients with lower BMI. We also cannot completely exclude that some patients took some micronutrients supplements and denied to declare it. Finally, determination of plasma concentrations in the routine setting is the only practical approach, but do not reflect the total body pool of these micronutrients, which is reduced proportionally to the reduction of fat mass and fat-free mass.

## 5. Conclusions

At initial evaluation of patients with AN of intermediate severity, at least one micronutrient deficiency is already observed in almost 50% of the patients. Other additional deficits are likely to appear during refeeding if appropriate micronutrients supplementation is not associated to macronutrient provision, which may lead to severe metabolic impairments and reduced efficiency of refeeding. Thus, micronutrient status must be closely monitored in AN patients during refeeding to prevent complications, improve nutritional outcomes and maybe also functional capacities and disease process through a reduction of oxidative stress.

## Figures and Tables

**Table 1:** Comparison of AN-R and AN-BP anthropometric features.

	AN-R (*n* = 91)	AN-BP (*n* = 62)	*t*-Test (*p*)
Mean age (years)	29.42 ± 11.4	27.1 ± 10.4	NS
Disease duration (years)	6.8 ± 7.6	8.4 ± 9.3	NS
BMI (kg/m^2^)	16.5 ± 2	18.8 ± 3.0	<0.05
FM (%)	18.7 ± 6.5	20.8 ± 5.9	*p* = 0.054
FFM (%)	80.6 ± 8.9	76.9 ± 12.6	<0.05

AN-R, anorexia nervosa—restricting subtype; AN-BP, anorexia nervosa—binge–purging subtype; BMI, body mass index; FM, fat mass; FFM, fat-free mass; NS, not significant. Values are means ± SD.

**Table 2:** Micronutrients mean values in AN-R and AN-BP patients.

	Mean Values	AN-R	AN-BP	*t*-Test (*p*)
Zn (9–17 µmol/L)	12.8 ± 1.9	13.04 ± 2.03	12.6 ± 1.7	NS
Cu (10–40 µmol/)	18.76 ± 5.8	18.40 ± 5.9	19.36 ± 5.5	NS
Ph (0.87–1.50 mmol/L)	1.19 ± 0.2	1.20 ± 0.17	1.17 ± 0.23	NS
Mg (0.75–1 mmol/L)	0.86 ± 0.64	0.87 ± 0.60	0.85 ± 0.72	NS
Ca^2+^ (2.15–2.55 mmol/L)	2.36 ± 0.92	2.35 ± 0.91	2.37 ± 0.09	NS
Se (0.90–1.65 µmol/L)	0.97 ± 0.21	0.99 ± 0.23	0.93 ± 0.18	NS
Vit E (7–17 mg/L)	11.89 ± 3.51	11.4 ± 3.07	12.68 ± 4.02	<0.05
B12 (141–489 pmol/L)	413.1 ± 193.1	426.3 ± 183.0	391.46 ± 177.7	NS
B9 (10.4–42.4 nmol/L)	19.42 ± 13.07	19.45 ± 10.59	19.38 ± 16.40	NS
Vit A (430–800 µg/L)	521.6 ± 164.6	511.6 ± 152.4	537.5 ± 182.7	NS

AN-R, anorexia nervosa—restricting subtype; AN-BP, anorexia nervosa—binge–purging subtype; NS, not significant. Values are means ± SD.

**Table 3:** Patients with micronutrient deficiency in AN-R and AN-BP.

	Deficiency % (*n*)	AN-R % (*n*)	AN-BP % (*n*)	*p* (Chi2)
Zn (9–17 µmol/L)	2.1 (3)	2.2 (2)	1.9 (1)	NS
Cu (10–40 µmol/L)	0 (0)	0 (0)	0 (0)	-
Ph (0.87–1.50 mmol/L)	3.5 (5)	3.5 (3)	3.6 (2)	NS
Mg (0.75–1 mmol/L)	3.0 (4)	1.2 (1)	6.0 (3)	NS
Ca^2+^ (2.15–2.55 mmol/L)	1.3 (2)	1.1 (1)	1.7 (1)	NS
Se (0.90–1.65 µmol/L)	40.6 (58)	40.0 (36)	41.5 (22)	NS
Vit E (7–17 mg/L)	1.5 (2)	1.2 (1)	1.9 (1)	NS
B12 (141–489 pmol/L)	2.2 (3)	2.4 (2)	1.9 (1)	NS
B9 (10.4–42.4 nmol/L)	15.9 (22)	14.1 (12)	18.9 (10)	NS
Vit A (430–800 µg/L)	32.8 (45)	35.7 (30)	28.3 (15)	NS
At least one oligoelement deficiency	43.7 (55)	43.0 (34)	44.7 (21)	NS
At least one vitamin deficiency	45.7 (59)	46.9 (38)	43.8 (21)	NS

AN-R, anorexia nervosa—restricting subtype; AN-BP, anorexia nervosa—binge–purging subtype; NS, not significant.

## Supplementary Materials

The following are available online at http://www.mdpi.com/2072-6643/9/3/225/s1.
