# Supplementary material for: Micronutrient Status in 153 Patients with Anorexia Nervosa
Source: Nutrients. 2017 Mar 2;9(3):225. doi: 10.3390/nu9030225 (PMC5372888; doi:10.3390/nu9030225)
Supplement: Supplementary file 1 [file nutrients-09-00225-s001.docx]

**Online supplement table:** Other biological parameters mean values in AN-R and AN-BP patients

AN-R, anorexia nervosa - restricting subtype; AN-BP, anorexia nervosa - binge-purging subtype; NS, not significant. Values are means ± SD.

|  | **Mean values** | **AN-R** | **AN-BP** | **T-test (p)** |
| --- | --- | --- | --- | --- |
| albumin [35 – 52 g/l] | 46.06 ± 4.6 | 46.53 ± 4.4 | 45.32 ± 4.9 | NS |
| transthyretin [0.20 – 0.45 g/l] | 0.26 ± 0.54 | 0.26 ± 0.05 | 0.25 ± 0.54 | NS |
| CRP [<5 mg/l] | 2.1 ± 9 | 1.19 ± 8.05 | 3.59 ± 8.2 | NS |
